# Light-driven transport of plasmonic nanoparticles on demand

**DOI:** 10.1038/srep33729

**Published:** 2016-09-20

**Authors:** José A. Rodrigo, Tatiana Alieva

**Affiliations:** 1 Universidad Complutense de Madrid, Facultad de Ciencias Fisicas, Ciudad Universitaria s/n, Madrid 28040, Spain

## Abstract

Laser traps provide contactless manipulation of plasmonic nanoparticles (NPs) boosting the development of numerous applications in science and technology. The known trapping configurations allow immobilizing and moving single NPs or assembling them, but they are not suitable for massive optical transport of NPs along arbitrary trajectories. Here, we address this challenging problem and demonstrate that it can be handled by exploiting phase gradients forces to both confine and propel the NPs. The developed optical manipulation tool allows for programmable transport routing of NPs to around, surround or impact on objects in the host environment. An additional advantage is that the proposed confinement mechanism works for off-resonant but also resonant NPs paving the way for transport with simultaneous heating, which is of interest for targeted drug delivery and nanolithography. These findings are highly relevant to many technological applications including micro/nano-fabrication, micro-robotics and biomedicine.

Metal plasmonic NPs (e.g. silver and gold) have attracted increased attention due to their peculiar properties^1^,^2^,^3^. Their optical response can be tuned in the visible and infrared spectral range as a function of the NP shape and size^2^,^3^. These NPs strongly absorb and scatter light in the spectral region near to their localized surface plasmon resonance (LSPR), and therefore, can be applied as heat nanosources for lithography^4^,^5^, photoacustic imaging^6^, photothermal therapy^7^,^8^, etc. In the last years, the familiar point-like laser traps exploiting intensity gradient forces have opened the door to developing all-optical nanofabrication^1^,^2^,^3^,^4^ of plasmonic structures required in photonic technologies^9^. However, such traps have reduced functionality because only a few plasmonic NPs can simultaneously be trapped and their manipulation requires shifting the focal spot accordingly^10^,^11^. These limitations make challenging massive optical transport of NPs. Moreover, point-like 3D traps are restricted to off-resonant wavelengths on the red-detuned side of the LSPR where the attractive intensity gradient forces have to be sufficiently strong to compensate repulsive scattering forces of light^2^,^3^,^10^. This makes difficult transport with simultaneous NPs heating, which is beneficial for different applications such as plasmonic assisted lithography^4^,^5^, photothermal therapy and drug delivery^12^,^13^, to name a few. An alternative technique consists of shaping both the intensity and phase of the trapping beam. Specifically, the high intensity gradient forces of the designed beam provide the particle confinement according to the required transport route, while the scattering forces associated with the beam’s transverse phase gradients^14^,^15^,^16^,^17^,^18^ allow for propelling the particle along the path. This approach has experimentally been demonstrated in recent works for 3D trapping of colloidal dielectric microparticles along circles and lines^14^, and arbitrary closed and open curves^18^. It has also been reported that a circular Gaussian vortex trap can set a plasmonic gold particle of 400 nm into fast rotation^19^. Nevertheless, Gaussian vortex trapping beams are also not suited for optical transport because they do not provide independent control of the trajectory size and speed of the particle^15^,^19^,^20^,^21^. So far the NP motion caused by driving phase gradient forces has only been shown in the case of a line trap for assembling of 150 nm silver particles^22^. In the latter work the NPs were confined against the coversilp glass because for the considered laser wavelength the axial intensity gradient force is repulsive.

Optical confinement and transport of numerous plasmonic NPs and nanoscopic structures (both resonant and off-resonant) is a crucial task in the mentioned applications as well as in a large variety of plasmonic-based technologies. This requires transport along arbitrary trajectories, stable confinement, independent control of the trajectory size and particle motion according to the considered application. In this work we present an optical manipulation tool exploiting only phase gradient forces providing an innovative solution to this challenging problem that is envisioned to assist such a technological development. The proposed tool enables confinement and programmable transport routing (including obstacle avoidance) of plasmonic nanostructures that paves the way to exploit their extraordinary capabilities. This is well suited for transport of off-resonant but also resonant NPs providing simultaneous heating that is useful for photothermal therapy, drug delivery and lithography. The trajectory can be open or closed, however here, as an example, we have considered several closed loop trajectories allowing for continuous transportation of the NPs. This kind of optical transportation can be applied for selective mixing of different types of NPs stored in separated reservoirs, for micro-pumping in optofluidics, and be used for shape-adaptive cell heating, etc.

## Results

**Figure 1:**
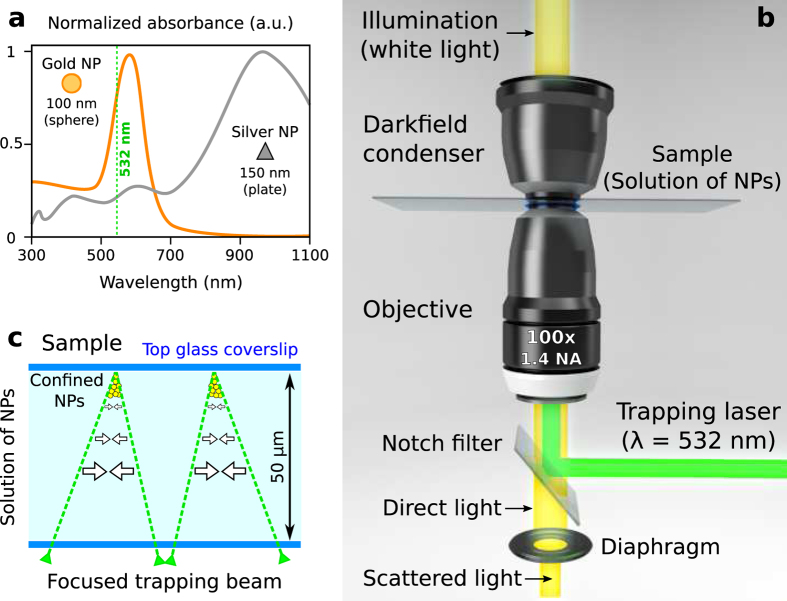
(**a**) Spectral absorbance of silver and gold NPs. The trapping laser was focused over the sample (colloidal solution of NPs) as sketched in (**b**). Dark-field illumination was applied to image the NPs by using the same objective lens required for the focusing of the trapping beam. (**c**) The NPs are confined (by transverse phase gradient forces, white arrows) near the top glass coverslip enclosing the sample (thickness of 50 *μ*m).

In contrast to other laser traps, the proposed confinement mechanism exploits transverse phase gradient forces^14^,^15^,^17^ that allows working with resonant and off-resonant wavelengths on both red/blue-detuned sides of the LSPR. The propelling mechanism is also governed by phase gradients independently prescribed along the curve^18^,^23^ that provides speed control of the particles without altering the size and shape of the trajectory. We explain and experimentally demonstrate how to exploit these mechanisms for programmable optical transport routing of silver and gold NPs along curved paths that can be *in-situ* tailored to around, surround or impact on objects present in the environment. Specifically, we have considered colloidal silver NPs of 150 nm (10 nm thick triangular plate, LSPR at 950 nm) and gold NPs of 100 nm (sphere, LSPR at 570 nm). The laser wavelength was 532 nm, which is on the blue-detuned side near the LSPR of gold NPs. In contrast, this wavelength being far from the LSPR of the silver NPs allows avoiding significant optical heating, see Fig. 1(a). Dark field illumination enables imaging the NPs due to the resulting scattered light collected through the same microscope objective focusing the trapping beam over the top glass coverslip, as sketched in Fig. 1(b,c).

As it has been recognized since Kepler’s *De Cometis* the light radiation pressure pushes objects along the beam propagation direction yielding the deflection of the comet tails pointing away from the sun. It is also known^14^,^15^,^17^ that the phase gradients orthogonal to the beam propagation direction redirect part of the light radiation pressure to exert forces able to propel the particles in the transverse plane. The familiar Gaussian vortex beam able to to set particles into rotation is a well-known example of this phenomenon. In our case, the transverse phase gradient forces of the light curve are described as 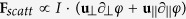, where *I* and *φ* are the intensity and phase distributions of the field with 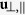 being normal and tangent vectors to the curve (see Methods). While the propelling force 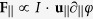 is responsible for the transport of the particles along the curve^14^,^15^,^18^, here we show that the perpendicular one ***F***_⊥_ ∝ *I* ⋅ ***u***_⊥_∂_⊥_*φ* governs the confinement within a toroidal channel created before the focusing plane of the curve. The force ***F***_⊥_ changes its sign after this plane and therefore expels the NPs from the channel. Thus, the 2D confinement is possible before this focusing plane when the upward axial NP motion is restricted by the top coverslip. This kind of confinement is the only option when using laser wavelengths on the blue and red sides of the LSPR for which the intensity gradient forces are repulsive or too weak. Nevertheless, for near-resonant wavelengths (on the red-detuned side) the attractive intensity gradient forces become stronger^2^,^10^ and the NPs could be stably trapped even in 3D within the curve^18^.

**Figure 2:**
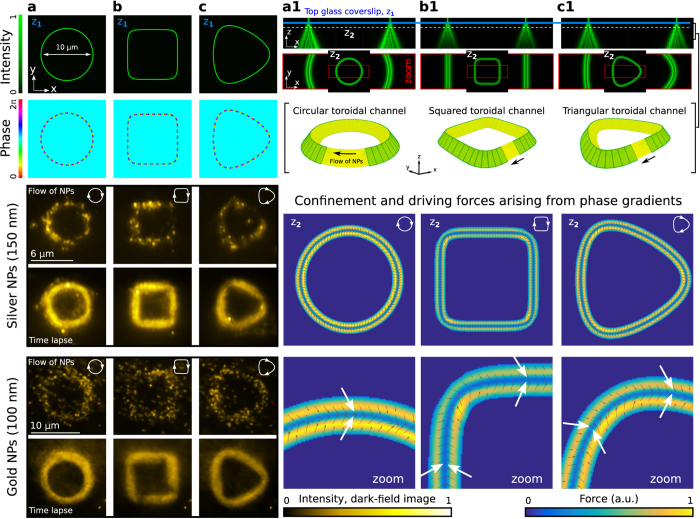
Top panel shows the intensity and phase of the laser trap focused (plane *z*_1_) into a circle (**a**), square (**b**) and triangle (**c**). Focusing profiles in the XZ plane for each case are shown in (a1), (b1) and (c1), correspondingly. A zoom inset of the intensity distribution at the plane *z*_2_ is displayed for each case in (a1), (b1) and (c1), for illustrating the shape of the toroidal channel. The corresponding distribution (vector field and modulus) of the confinement and driving forces (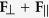) are displayed in the bottom panel of (a1), (b1) and (c1). A rotating flow of NPs confined within the toroidal channel is shown for each trap shape in the bottom panel in (**a–c**). Time lapse images of the flow are also displayed. In the case of the circle the considered values of topological charge are *l* = 15 (with radius 3 *μ*m) for silver NPs and *l* = 30 (with radius 5 *μ*m) for gold NPs. These values of charge *l* have also been applied for the square and triangle, correspondingly. The flow of NPs rotates clockwise for *l* > 0 and anticlockwise for *l* < 0, see Supplementary video 1 and 2.

To illustrate the confinement and propulsion mechanism, let us first consider three basic trajectories: a circle, square and triangle. The intensity and phase of the focused beam have been prescribed (see Methods) along these curves as displayed in Fig. 2(a–c), respectively. Specifically, the circle trap has a vortex structure exp(i*lθ*), Fig. 2(a), with *l* being the topological charge and *θ* the azimuthal angle^14^,^18^. The phase gradient forces ***F***_⊥_ of the trap (arising from the strong focusing of the curved beam) confine the NPs within a toroidal channel, whereas 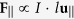 (arising from the vortex phase) yield a traveling flow of NPs along the channel, see Fig. 2(a–a1) and Supplementary video 1 and 2. The peak speed of the particles increases with the charge |*l*| because the driving force 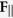 is proportional to *l*^14^. This confinement and transport mechanism also governs the NPs in other toroidal channel shapes, e.g.: square Fig. 2(b) and triangle Fig. 2(c). The corresponding distributions of the transverse forces 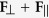, displayed in the bottom panel of Fig. 2(a1–c1), have been numerically calculated to confirm the experimental results. Note that such a NP confinement mechanism cannot be exploited in the case of a Gaussian vortex trap (blue-detuned) because the dominant repulsive forces ***F***_⊥_ are oriented towards its dark center, where the rotation of NPs has been observed^24^.

The flow of NPs is displayed in the bottom panel of Fig. 2(a–c) and its shape can be reconfigured in real time preserving the confined particles as shown in Supplementary video 1 (silver NPs) and video 2 (gold NPs). A time lapse image has been created by summing the recorded frames for each case in order to underline the shape of the flow, see Fig. 2(a–c), whose well-defined form confirms the strong confinement of the NPs even in the corners of the square and triangle. Easily switchable clockwise (*l* > 0) and anticlockwise (*l* < 0) rotating flows are demonstrated in Supplementary video 2. To estimate the rotation rate of the NPs, a particle tracking algorithm can be applied. Nevertheless, this is a challenging computational task due to the large number of confined NPs. Therefore, we have estimated the rotation rate by studying the impact of the considered circular flow of gold NPs with an obstacle, see Supplementary Information and video 4. After the impact a remaining flow of NPs vanished after ~200 ms, that gives an estimated rotation rate of about 2.5 Hz.

**Figure 3:**
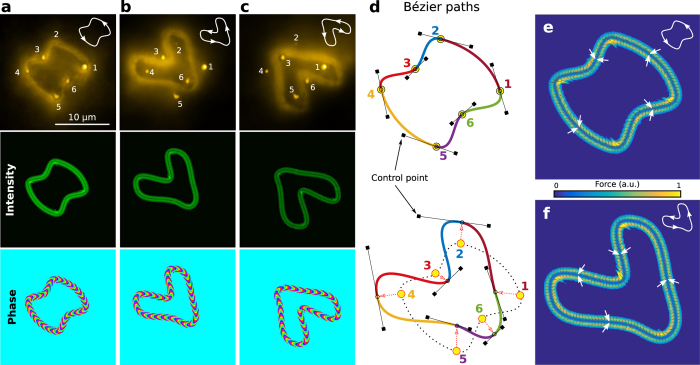
(**a**) Gold NPs transported (time-lapse flow image) along a curved Bézier path passing through six target objects (small clusters of gold NPs attached to the glass coverslip), see Supplementary video 3. The intensity and phase (charge *l* = −30) of the trapping beam were reconfigured to avoid these objects as it is shown in the bottom panel of (**b,c**), correspondingly. (**d**) Sketch of the considered automatic path-finding approach based on several Bézier curves (in this case six cubic splines governed by control points) indicated with different colors, see Methods. (**e,f**) Distributions of the corresponding confinement and driving forces (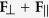) acting on the particles.

Real life applications require solving more complex NPs transport problems which are similar to robotic motion planning. It consists of detecting the positions of the targets to delivery the NPs and/or obstacles to avoid, and then designing the route and shaping the laser trap accordingly. The curve describing the route has to be smooth enough and the driving forces 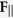 being uniform to avoid particle traffic jam. In the following examples, we demonstrate that this can automatically be achieved almost in real time. Six small clusters of NPs attached to the coverslip have been used to mimic targets or obstacles as displayed in Fig. 3(a–c), respectively. An automatic path-finding method based on Bézier curves (cubic splines^25^) has been applied to design the smooth route for each case, see Fig. 3(d) and Methods. As in the previous examples displayed in Fig. 2 the trapping beam has easily been shaped along these routes, see bottom panel of Fig. 3(a–c) where the intensity and phase in the trapping region are shown. The flow shape and motion of the NPs (Supplementary video 3) are in good agreement with the expected trajectories and force distributions, see Fig. 3(e,f), confirming the strong confinement and programmable transport in spite of the complex route shape.

## Discussion

The considered experiments underline the versatility and high performance of the proposed optical manipulation technique. This allows for guiding metal NPs along tailored trajectories for interaction with objects (e.g. vesicles and cells), exploiting off-resonant but also resonant laser wavelengths for simultaneous NP heating. Optical forces combined with simultaneous heating can push gold NPs inside vesicles^12^ or attach the NPs to the cell membrane yielding nanopores useful for drug delivery^13^. We envision that the presented optical manipulation tool could also play an important role in the future development of plasmonic nanotechnologies and applications adding promising functionality yet to exploit.

## Methods

### Confinement and driving forces on metal NPs in the dipole approximation

A dipolar NP with size *a* below the laser wavelength 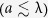 experiences time averaged radiation-induced forces^2^,^17^


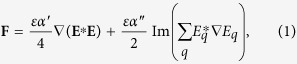


where: *q* = *x*, *y*, *z* is a placeholder for the coordinates, *α*(*λ*) = *α*′(*λ*) + i*α*′′(*λ*) is the particle polarizability while *ε* is the permittivity of the surrounding medium, and ***E*** is the electric field of the focused laser beam (trap). This total force splits into two contributions, the intensity gradient force proportional to *α*′ and the scattering force proportional to the dissipative part (*α*′′). Note that *α*′′ is always positive, while *α*′ can be negative near the LSPR (on the blue side). In the considered optical trap the laser beam is linearly polarized and the field can be written in paraxial approximation as *E*(*x*, *y*) = |*E*(*x*, *y*)|*e*^i*φ*(*x*,*y*)^, near to the focal plane of the focusing lens. Thus, the particle experiences traverse scattering forces:


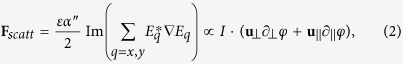


where *I* = |*E*(*x*, *y*)|^2^ and *φ* are the intensity and phase distributions of the field with 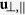 being normal and tangent vectors to the curve. Therefore, the transverse scattering forces in the normal and tangential directions naturally arise from the phase gradients along such directions. The role played by phase gradients to control the radiation pressure was previously underlined in refs 14 and 17. In our case, 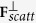 can confine the plasmonic NPs within a toroidal channel, while 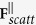 control their motion along the considered curve. The spatial distributions of these forces (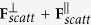) have been displayed in the bottom panel of Fig. 2(a1–c1) and in Fig. 3(e–f), for the considered curved traps. We recall that 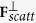 depends on the strong focusing of the beam, thus an objective lens with high numerical aperture (e.g. 1.4 NA) is preferred for stable confinement. Although for a high NA objective lens a more realistic model of the trapping beam is required^15^, the considered paraxial approximation provides a good qualitative description of the transverse forces acting on the particle well supported by the experimental results.

### Beam shaping technique

The laser traps have been created by focusing the following beam


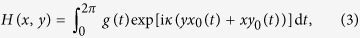


over the sample. This expression –encoded as a hologram into the spatial light modulator (SLM) as reported in ref. 23 –prescribes the intensity and phase of the trapping beam along arbitrary curves, where: 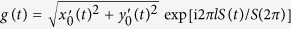, *κ* = 2*π*/*λ* f with f being the focal length of the focusing lens, and (*x*_0_(*t*), *y*_0_(*t*)) are the Cartesian coordinates of the parametrized curve. The function 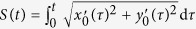 sets a uniform phase gradient along the curve. In this work we have considered closed parametric curves –circle (*x*_0_(*t*) + i*y*_0_(*t*) = *R*_0_ exp(i*t*)) of radius *R*_0_, square (*x*_0_(*t*) + i*y*_0_(*t*) = *R*_0_(−3 exp(i*t*) + 7 exp(−i3*t*)/20)), and triangle (*x*_0_(*t*) + i*y*_0_(*t*) = *R*_0_(−3 exp(i*t*) + 7 exp(−i2*t*)/20))− but also smooth paths constructed by using several Bézier curves (parametric cubic splines) as shown in Fig. 3(d). Note that open curves can also be applied if needed^23^. We underline that the integral *H*(*x*, *y*) has been numerically calculated (MATLAB) in about 10 seconds.

### Experimental implementation

The SLM (Holoeye PLUTO, pixel size of 8 *μ*m) was illuminated by a collimated laser beam (Laser Quantum, Ventus, *λ* = 532 nm, 1.5 W, linearly polarized) and the resulting beam relayed into the back aperture of the focusing lens: microscope objective lens (Olympus UPLSAPO, 1.4 NA, 100×, oil immersion *n* = 1.56 from Cargille Labs Series A^18^). The power of the laser beam was ~170 mW at the back aperture of the objective lens. The particles were observed under white light illumination (high power LED, SugarCube Ultra) by using an oil immersion dark-field condenser (Nikon, 1.4 NA). The dark-field image of the NPs was recorded by a sCMOS camera (Hamamatsu, Orca Flash 4.0, 16-bit gray-level, pixel size of 6.5 *μ*m) at 20 frames per second with exposure time of 10 ms. Note that a Notch filter (Semrock, dichroic beamspliter for 532 nm) redirected the trapping beam into the objective lens, that prevents saturating the camera by back-scattered laser light.

### Path-finding method based on Bézier curves

The transport route of the NPs can be created by using analytic parametric curves such as the circle and square. In practice, however, it is required much complex shapes tailored to the considered transport problem to guide the NPs towards certain targets and avoid obstacles. The proposed path-finding method creates a route by using several Bézier curves (cubic splines) whose shapes are governed by control points as sketched in Fig. 3(d). In our case, such control points are calculated using the method proposed in ref. 25, which automatically gives the slope and tension of the spline to be manually specified instead. The first step of the path-finding algorithm consists of detecting the position of the targets and then use them as reference points. The Bézier curves connect adjacent reference points as in the case of Fig. 3(a). On the other hand, the reference points are shifted creating new Bézier curves to avoid the obstacles. For example, in Fig. 3(b) only the obstacles in even positions are encircled or viceversa as in Fig. 3(c). To illustrate this procedure, the bottom panel of Fig. 3(d) displays the Béizer path created to avoid the six obstacles by using six shifted reference points.

### Sample preparation

The sample was enclosed into a chamber made by attaching two glass coverslip (thickness 0.17 mm). A Scotch tape (thickness ~50 *μ*m) was used as spacer between the coverslips. The NPs were filled into the sample cell directly from the aqueous solution provided by the manufacturer: 150 nm silver NPs (NanoComposix Inc., 10 nm thick triangular plates, PVP coated, Lott. JMW1340) and 100 nm gold NPs (Sigma-Aldrich, citrate stabilized Au spheres, 742031, Lott. MKBS6913V).

## Additional Information

**How to cite this article**: Rodrigo, J. A. and Alieva, T. Light-driven transport of plasmonic nanoparticles on demand. *Sci. Rep.* **6**, 33729; doi: 10.1038/srep33729 (2016).

## Supplementary Material

Supplementary Video S1

Supplementary Video S2

Supplementary Video S3

Supplementary Video S4

Supplementary Information
