# Supplementary material for: Light-driven transport of plasmonic nanoparticles on demand
Source: Sci Rep. 2016 Sep 20;6:33729. doi: 10.1038/srep33729 (PMC5028719; doi:10.1038/srep33729)
Supplement: Supplementary Information [file srep33729-s5.pdf]

Supplementary Information for:
"Light-driven transport of plasmonic nanoparticles on demand"

José A. Rodrigo* and Tatiana Alieva
Universidad Complutense de Madrid, Facultad de Ciencias Físicas, Ciudad Universitaria s/n, Madrid 28040, Spain

ROTATION RATE AND IMPACT OF THE FLOW OF METAL NANOPARTICLES WITH AN OBSTACLE

A clockwise ($l > 0$) and anticlockwise ($l < 0$) rotating flow of NPs can be configured in all the considered shapes. To illustrate this fact and the potential application of these traps for manipulation of metal microparticles, we have confined a small cluster (size $\sim 1\,\mu$m) of gold NPs into the laser traps as observed in Supplementary video 2. This cluster has been created by a laser beam focused (point-like) over the top coverslip to melt several gold NPs due to light absorption. The rotation rate of the cluster confined within the channel (with the charge $|l| = 30$) is 0.8 Hz (circle), 0.25 Hz (square) and 0.5 Hz (triangle). To estimate the rotation rate of the NPs, a particle tracking algorithm can be applied. Nevertheless, this is challenging computational task due to the large number of confined NPs. Therefore, we have conceived an additional experiment to estimate the rotation rate by studying the impact of the rotating flow of NPs with an obstacle. In particular, the confined flow of gold NPs was transported towards an obstacle created by melting gold NPs into a cluster (size $\sim 3\,\mu$m) attached to the top coversilp, see Supplementary video 4 and Fig. S1. Before their impact ($t = 0 - 0.4$ s), shown in Fig. S1(a), the rotating flow of NPs is stable, see also the time lapse image of Fig. S1(b). After the impact ($t \sim 1$ s) a remaining flow of NPs is only visible along half of the original circle because most of the NPs escape. This remaining flow of NPs vanished after ~ 200 ms, that gives an estimated rotation rate of about 2.5 Hz. The collision course can be changed by setting the opposite charge l as observed in Supplementary video 4. Interestingly, a regular flow of NPs is observed when the obstacle is placed in the center of the trap as shown in Fig. S1(c). This also proves that a rotating flow of NPs can be set around objects.

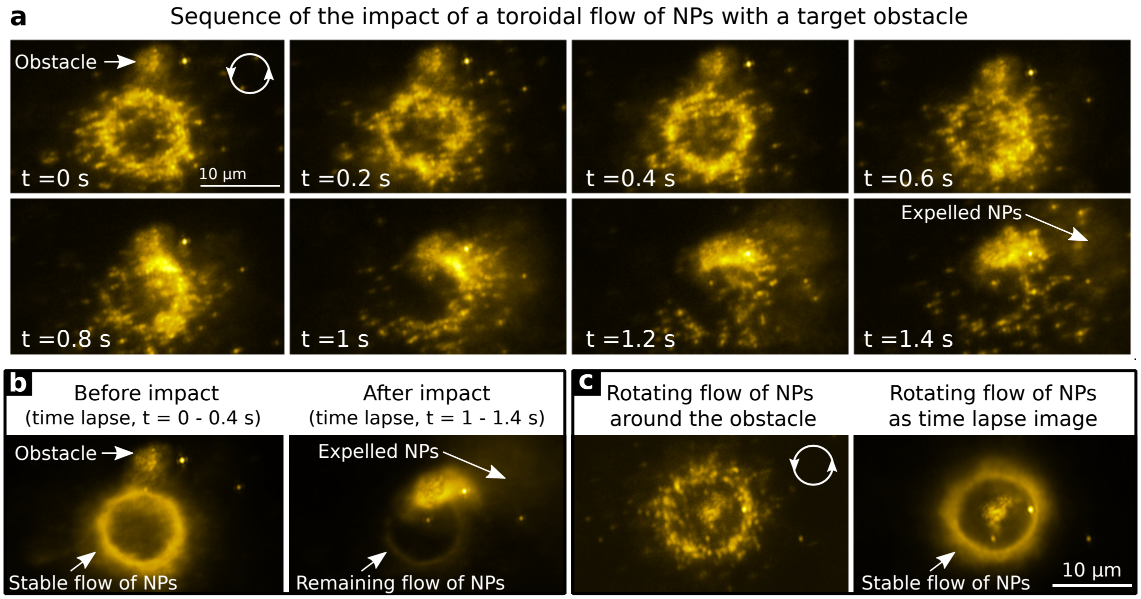

Figure S1. **a**, The rotating flow of gold NPs is transported towards an obstacle (cluster of gold NPs attached to the glass coverslip). The sequence shows the interaction between them recorded in Supplementary video 4. **b**, Before the impact the rotating flow of gold NPs (time lapse image) is stable but after the impact ($t \sim 1$ s) it is only observed a remaining flow (see also panel **a**) because most of the NPs escape. The collision course can be changed by setting the opposite charge l, thus allows for hitting both sides of the obstacle. **c**, Stable rotating flow of NPs observed when the trap encircles the obstacle. See Supplementary video 4.

* jarmar@fis.ucm.es

We recall that the translation of the sample preserves the confined rotating flow of NPs. This is observed in Supplementary video 4 for gold NPs but also in Supplementary video 1 for the case of silver NPs. Thus, the confined particles can be transported and delivered to different regions of the sample if needed. Another important fact is that the shape and size of the trap can be changed in real time (a few milliseconds) to generate different geometries of the rotating flow of NPs, preserving the particle confinement, see: Supplementary video 1 (silver NPs) and video 2 and 3 (gold NPs).
